# Validation of novel conditional ligands and large-scale detection of antigen-specific T cells for H-2D^d^ and H-2K^d^

**DOI:** 10.1038/s41598-024-62938-8

**Published:** 2024-05-29

**Authors:** Trine Sundebo Meldgaard, Nadia Viborg, Sara Suarez Hernandez, Dario Vazquez Albacete, Tripti Tamhane, Sine Reker Hadrup

**Affiliations:** 1 Department of Health Technology, Section of Experimental and Translational Immunology, Technical University of Denmark, Kongens Lyngby, Denmark; 2 Present Address: Novo Nordisk, Copenhagen, Denmark; 3 Present Address: Evaxion Biotech, Hørsholm, Denmark; 4 Present Address: RIVM National Institute for Public Health and the Environment, Utrecht, The Netherlands; 5 Present Address: Novonesis, Copenhagen, Denmark

**Keywords:** MHC class I, Immunosurveillance, Cancer models

## Abstract

The UV-mediated peptide exchange has enabled the generation of multiple different MHC multimer specificities in parallel, surpassing tedious individual refolding of MHC molecules with peptide ligands. Murine models are acknowledged as an effective tool for preclinical research to advance our understanding of immunological mechanisms, with the potential translatability of key learnings from mouse models to the clinic. The common inbred mouse strain BALB/c is frequently used in immunological research. However, for the BALB/c histocompatibility (H)-2 alleles availability of conditional ligand has been limited. To overcome this challenge, we design and experimentally validate conditional ligands restricted to murine MHC class I alleles H2D^d^ and H2K^d^. In addition, we demonstrate the ability of the three H2^d^ molecules and two additional C57BL/6 H2^b^ molecules folded in-house with conditional ligands to generate fluorescently labeled peptide-H2 tetramers that allow staining of antigen-specific CD8+ T cells in splenocyte samples. Finally, we generate large peptide-H-2 multimer libraries with a DNA-barcode labeling system for high-throughput interrogation of CD8+ T cell specificity in murine splenocyte samples. Consequently, the described techniques will contribute to our understanding of the antigen-specific CD8+ T cell repertoire in murine preclinical models of various diseases.

## Introduction

Major histocompatibility complex (MHC) class I molecules display endogenous peptide products of proteasomal degradation on the surface of all nucleated cells. T cell-based immune surveillance relies on this presentation and the specific interaction between peptide-MHC (pMHC) and the T cell receptor (TCR), enabling cytotoxic T cells to identify and eliminate aberrant cells. Two decades of research have provided tools to identify, characterize and isolate antigen-specific T cells. In particular, multimerization and fluorochrome labeling of MHC I molecules have accelerated the understanding of pMHC-TCR interactions within infections, autoimmune diseases, and cancer, where CD8+ T cells play a pivotal role^1^. pMHC tetramers are commonly used in the field and can facilitate the detection of multiple different antigen-specific CD8+ T cells in parallel when e.g. combinatorial fluorescent labeling is used^2^.

An innovative contribution to the pMHC multimer space occurred when MHC protein was produced and refolded with a so-called conditional ligand (p*). A technique first described by Toebes et al.^3^, where an amino acid moiety in the T cell receptor-exposed site of a known MHC ligand is replaced with a non-natural amino acid (2‐nitrophenylglycine or 3‐amino‐3‐(2‐nitrophenyl)‐propionic acid) (the latter is used here and denoted “J”) that is cleavable upon exposure to 366 nm UV light. Upon UV light-mediated cleavage of the p*, the MHC binding groove is left empty and receptive to another ligand of choice. This UV-mediated exchange allows for the rapid and high-throughput generation of large panels of distinct pMHC specificities.

Since the development of pMHC tetramers and the introduction of the UV exchange technology, higher-order pMHC, multimerization, and high-throughput labeling systems have been developed. Recently, DNA barcode-labeled pMHC multimers were proven to allow large-scale detection of antigen-specific CD8+ T cells, with the possibility to screen samples for recognition of > 1000 different pMHC multimers simultaneously^4^. A technology that can contribute to uncover new T cell epitopes and understand pMHC-TCR interactions in a wide variety of diseases.

Preclinical models have proven important for acquiring a mechanistic understanding of immunological diseases and supporting the development and evaluation of new therapeutic interventions. Mouse models are frequently used in cancer research, where especially strategies to target cancer-specific mutation-derived neoepitopes are being extensively studied^5–8^. A number of syngeneic tumor models have been developed based on the inbred strains BALB/c and C57BL/6. These models represent cancer from a range of different tissue origins and show different levels of immunogenicity and treatment sensitivity^9^. There is a great need to enable the screening of murine samples in a high-throughput manner to identify CD8+ T cells responsive to e.g. tumor neo-epitopes predicted via mutational mapping and following in silico-based prediction of the MHC I binding characteristics. Such predictive strategies lead to the identification of large peptide libraries, for which limited knowledge is currently available governing the rules that determine T cell immunity. For the generation of large libraries of pMHC complexes, several peptide exchange technologies has been developed such as chaperone-mediated peptide exchange, temperature induced exchange^10–12^, and the most widely used UV-mediated peptide exchange. However, while there are several published descriptions of UV-cleavable H-2 ligands (here denoted p*), these have been lacking for the BALB/c H-2^d^ alleles H-2D^d^ and H-2K^d^^13–15^.

In this study, we design and validate p* for murine MHC I alleles H-2D^d^ and H-2K^d^. We use these together with H-2L^d^ and two C57BL/6 alleles H-2D^b^ and H-2 Kb to setup a DNA barcode labeled peptide-H-2 (p*H-2) multimer library, consisting of 120 pH-2 multimer complexes and use this to screen murine splenocyte samples in a high-throughput manner for detection of antigen-specific CD8+ T cells.

## Results

### Design of p* for murine MHC alleles H-2Dd and H-2Kd

MHC molecules bind peptides based on certain amino-acid residues, in given positions (anchor positions). The binding motif is unique for each MHC haplotype, and peptide binding is essential for the stability of the molecule. To retain the MHC binding capacity, UV-ligands are generated by replacing one of the nonanchor residues with by 3‐amino‐3‐(2‐nitrophenyl)‐propionic acid (here donoted “J”). The effect on protein stability can be accessed via structural remodeling or by analyzing the binding affinity using NetH2pan^14–17^. We designed and tested p* for H-2D^d^ and H-2K^d^ in-house, based on well described, high-affinity ligands; RGPGRAFVTI from HIV Env gp160 antigen and IYSTVASSL from influenza HA antigen, respectively For both H-2D^d^ and H-2K^d^, we introduced the UV-cleavable “J” amino acid in the TCR-facing part of the peptide sequence. For H-2Dd the key positions and preferences for peptide binding include: glycine (G) at P2, proline (P) at P3, and isoleucine (I) at P9 or P10 along with a positive charge (e.g. R) at P5^18^. Hence, we introduced the “J” amino acid by insertion at P7 (Table 1). For H-2Kd the key positions and preferences for peptide binding include: tyrosine (Y) at P2, leucine (L) at P9 or P10 along with an uncharged residue at P5 (e.g. V)^19,20^. Therefore, we introduced the “J” amino acid by substitution at position 6 (Table 1). H-2Dd and H-2Kd peptide binding motifs are visualized in Supplementary Fig. 1a and b respectively, generated based on ligand data from^21–25^. The H2 binding affinity for each UV ligand was predicted using NetH2pan and reported in this study as a %rank score (Table 1). As per NetH2pan recommendations, strong H-2 binders are generally considered to have a %rank score of < 0.5, and weak MHC binders have a %rank score of < 2. We aim for UV-cleavable ligands to be strong binders.

**Table 1:** Overview of investigated H-2 alleles and relevant epitopes.

Mouse strain	MHC class I allele	Conditional UV ligands (%rank score)	Specific/immunization epitopes (%rank score)
*C57BL/6*	H-2D^b^	ASNEN-**J**-ETM^10^ *(0.0026)*	RAHYNIVTF (HPV16 E7_49-57_)^34^ *(0.0592)*
	H-2 Kb	FAPGNY-**J**-AL^11^ *(0.3095)*	SIINFEKL (Ovalbumin_257-264_)^38^ *(0.0027)*
*BALB/c*	H-2D^d^	RGPGRA-**J**-VTI *(0.0347)*	RGPGRAFVTI (HIV Env gp160_311-320_)^36^ *(0.0081)*
	H-2K^d^	IYSTV-**J**-SSL *(0.0149)*	IYSTVASSL (Influenza HA_518-526_) *(0.0077)*
	H-2L^d^	YPNVNIH-**J**-F^12^ *(0.01)*	RPQASGVYM (LCMV NP_118-126_)^37^ *(0.0586)*

### UV exchangeable MHC monomers were produced for H-2b and H-2d MHC alleles

**Figure 1:**
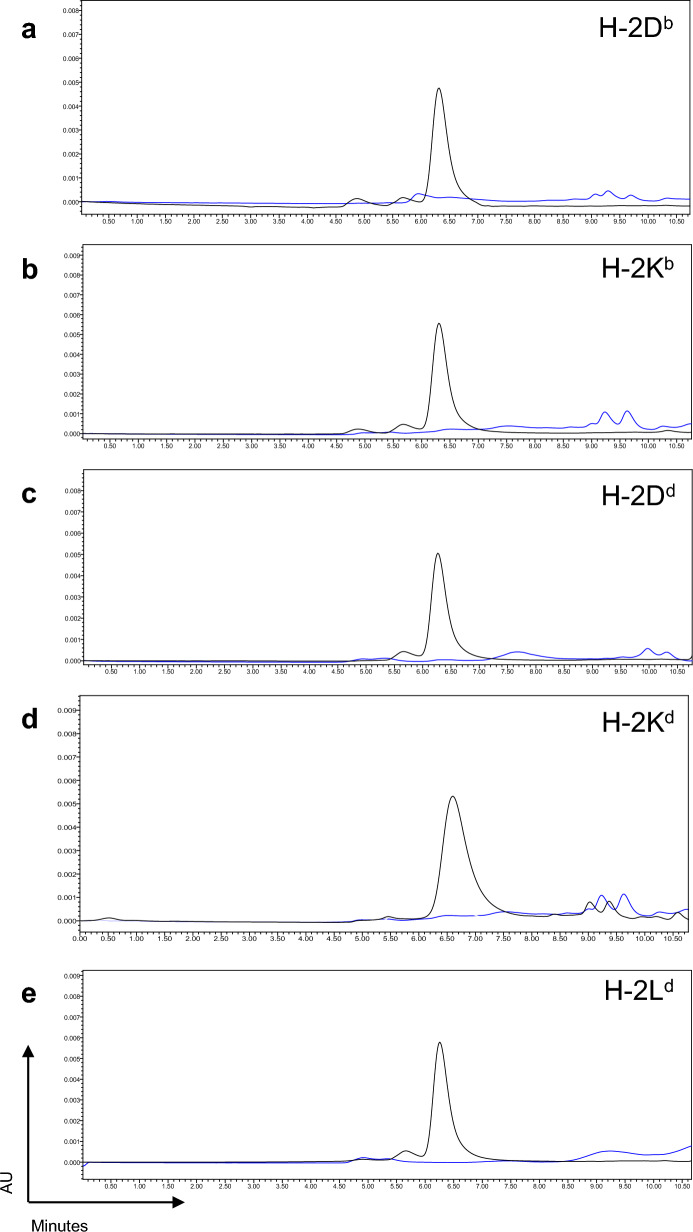
Murine MHC molecules folded with photocleavable peptides. HPLC chromatograms of purified MHC molecules folded using a MHC-specific photoclevable-peptide (black), and compared after incubation under UV-light for one hour showing complete dissociation of p*MHC complexes (blue) for H-2D^b^ (**a**), H-2 Kb (**b**), H-2D^d^ (**c**), H-2K^d^ (**d**), and H-2L^d^ (**e**) murine MHC molecules. Peak height corresponds to the amount of stable protein normalized for comparative runs on each plot.

Murine MHC class I molecules were produced in-house and folded with an MHC allele-specific UV cleavable p* (Table 1). The final protein yield depends on the folding setup and varies among alleles (Supplementary table 1). MHC molecule is stable only in the form of the trimer (p* + heavy chain + β2m). Degradation of p* is important for the exchange with the peptide of interest upon UV exposure. We validated the cleavage and dissociation of the p* from the binding groove which results in MHC disintegration in the absence of a rescue peptide, by size exclusion chromatography (Fig. 1 a-e).

**Figure 2:**
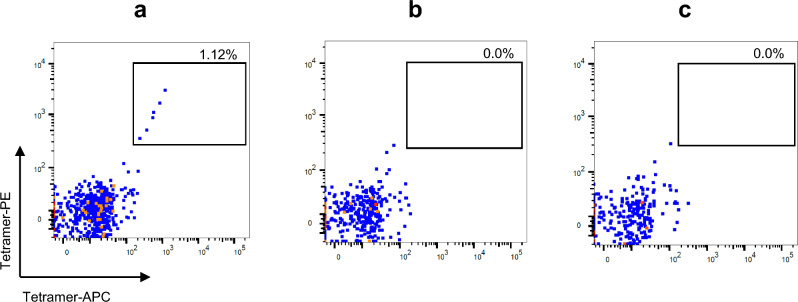
The assessment of peptide exchange efficiency upon UV exposure and effect of p* on T cell staining. H-2 Kb specific OT-1 cells were spiked into a human buffy coat and stained with H-2 Kb tetramers, based on dual color staining using PE and APC labeled tetramers, assembled using following monomers—(**a**) SIINFEKL loaded monomers generated after UV exchange of p*H-2 Kb, (**b**) UV light exposed p*H-2 Kb without SIINFEKL, and (**c**) only p*H-2 Kb. PE -APC-labelled tetramer-positive cells were gated from the mCD3 population. The frequency of pH-2 tetramer-specific cells is given in the top right corner of each plot.

**Figure 3:**
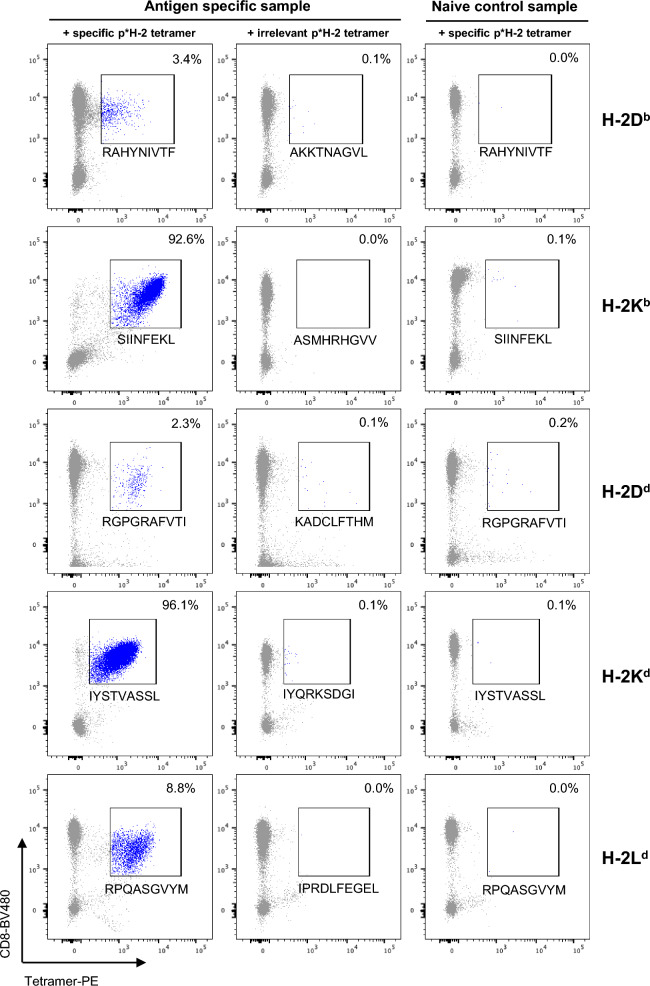
Detection of antigen specific CD8+ T cells via PE-labelled p*H-2 tetramers Successful UV-mediated peptide exchange and pH-2 tetramer assembly was validated for all H-2 alleles by tetramer staining (PE fluorophore label). Antigen specific splenocytes were stained with specific peptide tetramers and tetramers exchanged with irrelevant peptides to examine for unspecific background from the tetramer reagents. Naïve control splenocytes of matching strain were stained with specific peptide tetramers as a negative control. The frequency of pH-2 tetramer specific CD8+ T cells out of total CD8+ T cells is given on the top right corner of each plot.

T cell staining of murine OT-1 T cells specific for H-2 Kb SIINFEKL, with flourescent H2-multimers, generated from either p* loaded H-2 Kb monomers, or H-2 Kb exposed to UV light in the absence of test peptide-SIINFEKL did not show any T cell specific binding; while the population of OT-1 T cells could be detected using H-2 Kb exchanged with SIINFEKL, which shows the efficient peptide exchange and no non-specific signal due to effect of UV cleavable p* (Fig. 2 and FACS gating strategy—Supplementary Fig. 2). In addition, T cell staining with the pMHC molecules prepared via this process indirectly proves the capacity for peptide loading and exchange of p* with the peptide of interest for each MHC allele upon UV light exposure. Thus, the H-2^d^ possesses the properties required for UV-mediated peptide exchange and large library H-2 generation (Fig. 3).

### Detection of antigen-specific CD8+ T cells in murine splenocyte samples via pH-2b and pH-2d

#### tetramer staining

To confirm the presence of antigen-specific CD8+ T cells in our murine splenocyte samples and validate the potential use of the newly designed UV ligands for H-2 monomers, we generated pH-2 monomers by UV exchange and multimerized these biotinylated pH-2 products using PE-conjugated streptavidin. Splenocyte samples from immunized or transgenic mice (from here on: antigen-specific mice) were stained with either 1) pH-2 tetramers exchanged with the specific peptides corresponding to the specificity induced by immunization or the transgenic TCR clone specificity (Table 1), or 2) pH-2 tetramers exchanged with an irrelevant peptide (predicted H-2^b^ and H-2^d^ ligand neo-peptides from syngeneic tumor cell lines, Supplementary Table 2). As controls, splenocyte samples from naïve C57BL/6 or BALB/c mice were stained with the same specific peptide tetramers as splenocytes from the antigen-specific mice.

By flow cytometry, we confirmed pH-2 tetramer specific CD8+ T cells present in all antigen-specific mouse samples (Fig. 3). Observed frequencies of antigen-specific CD8+ T cells varied, with the TCR transgenic mice (SIINFEKL/H-2 Kb and IYSTVASSL/H-2K^d^) having > 90% tetramer specific cells, and the immunized mice varying from 2.8% (RAHYNIVTF/H-2D^b^), 0.8% (RGPGRAFVTI/H-2D^d^), and 6.5% (RPQASGVYM/H-2L^d^) antigen-specific T cells. The different antigen-specific CD8+ T cell populations displayed differences in fluorescence intensity based on the tetramer binding, reflecting differences either in TCR surface expression level or TCR-pMHC affinity. Control staining of antigen-specific samples with irrelevant pH-2 tetramers or naïve splenocyte samples with specific pH-2 tetramers revealed that the pH-2 tetramers generate little to no unspecific background and display a pH-2-TCR restricted T cell interaction. FACS gating strategy for pH-2 tetramer stainings is shown in the (Supplementary Fig. 3). Thus, we have successfully generated pH-2^b^ and pH-2^d^ tetramers after UV mediated peptide exchange, and demonstrated staining of antigen-specific CD8+ T cells in relevant mouse splenocytes.

### Large-scale interrogation of CD8+ T cell specificity using DNA barcoded pH-2 multimer libraries

To evaluate the use of H-2^b^ and H-2^d^ for large-scale interrogation of CD8+ T cell specificity, we generated a p*H-2 multimer panel as previously described^4^. In brief, we performed UV-mediated peptide exchange in separate wells for each pH-2 monomers, and hereafter multimerized the pH-2 monomers on a PE-labeled polysaccharide dextran backbone individually coupled with a DNA barcode unique to each pH-2 specificity. Therefore, multiple DNA barcode pH-2 multimers can be pulled together as a single staining reagent. MHC multimers should be mixed just prior to T cell staining to avoid peptide-exchange among the pooled pH-2 multimers. We included five specific peptides corresponding to the specificity induced by immunization or the transgenic TCR clone specificity (RAHYNIVTF/H-2D^b^, SIINFEKL/H-2 Kb, RGPGRAFVTI/H-2D^d^, IYSTVASSL/H-2K^d^, and RPQASGVYM/H-2L^d^). For each H-2 allele, we furthermore included > 22 irrelevant control peptides, as previously mentioned. As an additional control, we included a multimerized form of the respective H-2 allele without UV-mediated exchange, thus containing the conditional UV-sensitive ligand, p*H-2 multimer. This yielded > 24 different specificities for each allele, adding up to a total pH-2 library of 24–120 different specificities. A list of the full panel can be found in Supplementary Table 1.

Antigen-specific splenocyte samples were stained with the pH-2 multimer library. Splenocytes from transgenic mice were spiked between 1 to 10% into naïve samples to reduce the frequency of specific CD8+ T cells, gating strategy for the pH-2 multimer library staining is shown in Supplementary Fig. 4. Additionally, antigen-specific samples were mixed, to generate a sample pool (termed “H-2 spike-in mix”), allowing the detection of all five different antigen-specific CD8+ T cell populations in a single one-pot approach, and stained either with the full pH-2 multimer panel (Supplementary Fig. 5b, gating strategy) or the full p*H-2 multimer panel but without specific pH-2 multimers (RAHYNIVTF/H-2D^b^, SIINFEKL/H-2 Kb, RGPGRAFVTI/H-2D^d^, IYSTVASSL/H-2K^d^, and RPQASGVYM/H-2L^d^) (Supplementary Fig. 5c, gating strategy). The gating strategy for this experiment is shown in Supplementary Fig. 5a. All samples were acquired by flow cytometry and CD8+ T cells binding to pH-2 multimers were sorted (all PE + cells), and the associated DNA barcodes were amplified by PCR to reveal the specificity of the CD8+ T cells by sequencing of the co-attached DNA barcodes. T cell responses were identified based on the enrichment of a given pH-2 barcode-labeled MHC multimer in the sorted T cell fraction.

**Figure 4:**
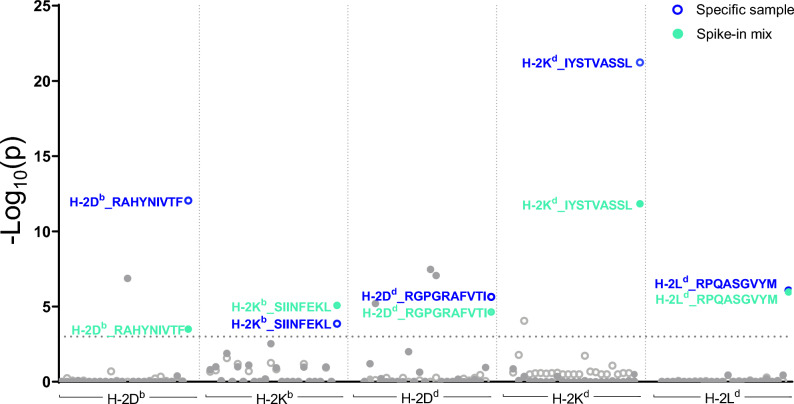
Detection of H-2 antigen specific CD8+ T cell responses via DNA barcode labeled pH-2 multimers. Screening for T cell recognition by DNA barcode-labeled pH-2 multimers in splenocytes from antigen specific mice with a library of 24–120 different pH-2 specificities (24–28 specificities per allele). Colored circles represent CD8+ T cell responses detected in H-2D^b^ RAHYNIVTF specific sample, H-2 Kb SIINFEKL specific sample H-2D^d^ RGPGRAFVTI specific sample, H-2K^d^ IYSTVASSL specific sample, H-2L^d^ RPQASGVYM specific sample (dark blue), or a spike-in mix of all specific H-2 samples (light green). All circles lying on the same vertical axis implies multiple CD8 + T cell responses to the same peptide across more than one splenocyte sample. Data plotted on the y-axis as –Log 10(p) of the relevant pH-2 associated DNA barcode, with a significance level of p < 0.001 (dotted line). The plots represent one or two separate experiments.

In agreement with the single pH-2 tetramer stainings, the large-scale screening with the total pool of 24–120 pMHC specificities identify the antigen-specific CD8 T-cells for which the mouse was immunized. For example, only the RAHYNIVTF/H-2D^b^-specific CD8+ T cells were detected in the RAHYNIVTF immunized mouse (Fig. 3). In the H-2 spike-in mix + sample, all five different antigen-specific CD8+ T cell populations were observed after sequencing, underlining the ability to detect the same responses even after dilution due to the mixing of samples (Fig. 4). The estimated frequency of multimer-specific T cells observed from the barcode-multimer analysis corresponds to the theoretical frequency of each multimer-specific T cell population in the spike-in-mix sample as shown in Supplementary Fig. 6. As a control for specificity, we evaluated both naïve control splenocytes (C57BL/6 and BALB/c) with the full pH-2 multimer panel and pH-2 spike-in mix sample with a pH-2 multimer panel not containing the five specific pH-2 multimers. None of the control antigen-specific CD8+ T cell populations were detected in these samples.

## Discussion

The data presented here, demonstrate for the first time, the design and use of p* for murine MHC class I alleles H-2D^d^ and H-2K^d^. These novel p* facilitate the generation of specific pH-2 multimers via UV-mediated peptide exchange, alongside p*H-2D^b^, p*H-2 Kb, and p*H-2L^d^ for which p* has previously been described. The UV-mediate peptide exchange technology enables the production of large libraries of pH-2 in parallel, overcoming the challenges and need for tedious individual folding of H-2 molecule with specific peptides. First, we were able to generate fluorescently labeled H-2 tetramers to stain murine H-2^b^ and H-2^d^ haplotype samples for single antigen-specific CD8+ T cell populations, obtained via peptide immunization or from TCR transgenic mice. Flow cytometric analysis confirmed the presence of specific CD8+ T cells in relevant antigen-specific splenocyte samples. Second, we used the UV mediated peptide exchange to generate a DNA barcode-labeled pH-2 multimer libraries containing 24–120 different pH-2 specificities. Flow cytometric sorting of multimer positive populations and recovery of DNA barcodes by sequencing corresponded to our findings from the tetramer stainings, i.e. antigen-specific CD8+ T cells were observed in antigen-specific splenocyte samples.

The high-throughput p*MHC multimer methodology has recently been used to describe human autoreactive CD8+ T cells in narcolepsy^26^, CD8+ T cells specific to novel overexpressed antigens in breast cancer^27^, and CD8+ T cell recognition towards SARS-CoV-2^28^. This technology enables the interrogation of samples for hundreds of pMHC specificities in parallel, which is particularly advantageous when sample material is sparse, as described previously^4^. Importantly, platforms like the one described here allow extensive screening of pMHC-TCR interactions that will contribute to the identification of T cell epitopes in various disease contexts, such as cancer and autoimmunity. The ability to identify such epitopes also in the murine disease models, based on the H-2 display as described here, can substantially improve the value of such murine models for preclinical evaluation within immunotherapy and vaccine development.

In cancer research, preclinical mouse models have proven valuable for the evaluation of new therapeutic approaches and for identifying immunological recognition of tumor cells. Syngeneic tumor models based on inbred strains C57BL/6 and BALB/c are being extensively used for the evaluation of immunotherapies, where particularly tumor neo-epitopes are considered therapeutically relevant targets. The in silico platforms that map tumor mutations and predict MHC binding peptides from the tumor peptidome generate vast output libraries of putative neo-epitopes. Combining the p*H-2 molecules with high-throughput multimer-based screening via the methodology described here, provides a tool to address the neo-epitope specific CD8+ T cell repertoire across tumor models and therapies. It has been established, that different syngeneic tumor models are not equally sensitive to immunotherapies^9,29^. Large-scale multimer analyses of the T cell repertoire will contribute to investigate the signatures of neo-epitope reactive T cells that favor immunotherapy responses.

In the present study, novel p* for H-2D^d^ and H-2K^d^ were designed and developed, and the data presented here add to the value of previously described ligands. In addition, the proof of concept of H-2^b^ and H-2^d^ multimers for large-scale detection of antigen-specific CD8+ T cells in murine samples will contribute to the field of epitope discovery and immune assessments going forward.

## Materials and Methods

### Expression and purification of murine MHC class I heavy chains and β2 microglobulin

A bacterial expression system was used to produce murine H-2 class I heavy chains (H-2D^d^, H-2K^d^, and H-2L^d^), and human β_2_m as described previously^30^. Briefly, proteins were produced in *Escherichia coli* Bl21(DE3) pLysS strain using pET series plasmids. Protein expression was induced by 0.5 mM isopropyl-beta-D-thiogalactopyranoside for 4 h. Inclusion bodies containing expressed proteins were lysed in lysis buffer (50 mM Tris·HCl (pH 8.0), 25% sucrose, 1 mM EDTA, and Lysozyme). Inclusion bodies were harvested by washing in detergent buffer (20 mM Tris–HCl, (pH 7.5), 200 mM NaCl, 1% NP40, and 1% Deoxycholic acid) followed by wash buffer (1 mM EDTA, 5% Triton X-100, 1 mM DTT). Next, inclusion bodies were dissolved in 8 M Urea buffer (8 M Urea, 50 mM K·HEPES pH 6.5 and 100 µM β-mercaptoethanol), and insoluble impurities were removed by centrifugation at 40,000xg for 20 min. The soluble fraction containing proteins was stored at − 80 °C until used for in vitro folding.

### In vitro folding and purification of murine MHC class I monomers

The in vitro folding of murine MHC class I molecules was performed using photocleavable ligand as described above and previously^3^. Conditional UV ligands were purchased and synthesized from Leiden University Medical Center peptide synthesis unit (LUMC) following previously described methods^31^ To setup the refolding reaction, heavy chains (1 µM) and β2m (2 µM) were diluted in a folding buffer composed of 0.1 M Tris pH 8.0, 500 mM L-Arginine-HCl, 2 mM EDTA, 0.5 mM oxidized glutathione and 5 mM reduced glutathione with 60 µM respective photocleavable peptide (H-2Db: ASNEN-J-ETM, H-2 Kb: FAPGNY-J-AL, H-2Dd: RGPGRA-J-VTI, H-2Kd: IYSTV-J-SSL, H-2Ld: YPNVNIH-J-F) (Table 1). After folding for 3–5 days at 4 °C, the folded protein was upconcentrated with a 10 kDa cut-off membrane filter (Vivaflow-200; Sartorius, Cat#VS0601) and biotinylated using BirA biotin-protein ligase standard reaction kit (Avidity, LLC- Aurora, Colorado). Finally, folded biotinylated monomer complexes were purified with size exclusion chromatography using HPLC (Waters Corporation, USA), and aliquots were stored at − 80 °C until further use.

### In vivo studies and splenocyte preparation

All animals were cared for following institutional guidelines. BALB/cJRj and C57BL/6JRj mice were acquired from Janvier Labs and housed at the Department of Health Technology, Technical University of Denmark. CL-4-TCR^32^ and OT-I-TCR^33^ transgenic mice were a kind gift from Dr. Ana Misslitz, Hannover Medical School, and Torsten Joeris from Lund University, respectively. To assess the binding of murine MHC class I monomers, the following in vivo studies were performed to induce reactive CD8+ T cells:

H-2Db specific T cells: C57BL/6JRj mice were injected with 50 µg each of two HPV16 E7 peptides (RAHYNIVTF^34^ and QAEPDRAHYNIVTFCCKCD^35^) formulated in CAF09b adjuvant (200 µg DDA, 40 µg MMG, 10 µg poly-IC, a kind gift from Dennis Christensen, Statens Serum Institut, Denmark). The mice were immunized three times intraperitoneally (i.p.) with a weekly interval, spleens were harvested 7 days post last immunization. H-2 Kb specific T cells: splenocytes from transgenic OT-I mice were harvested and spiked into splenocytes from naïve C57BL/6JRj mice. H-2Dd specific T cells: BALB/cJRj mice were injected with 80 µg HIV Env gp160 peptide (RGPGRAFVTI^36^) formulated in 30 µg poly-ICLC (Hiltonol^®^, gifted by Oncovir, Inc.). The mice were immunized four times i.p. with a weekly interval, spleens were harvested 7 days post last immunization. H-2Kd specific T cells: splenocytes from transgenic CL-4 mice were harvested and specific cells spiked into splenocytes from naïve BALB/cJRj mice. H-2Ld specific T cells: BALB/cJRj mice were injected with 80 µg LCMV NP peptide (RPQASGVYM^37^) formulated in 30 µg poly-ICLC. The mice were immunized four times i.p. with a weekly interval, spleens were harvested 7 days post last immunization.

Mice were euthanized by cervical dislocation and spleens were harvested and kept on a 4 °C medium (Roswell Park Memorial Institute (RPMI), Gibco RPMI 1640, Cat#72,400,054 and 10% Fetal Calf Serum (FCS), Gibco, Cat#10,500,064). Spleens were placed in a GentleMACS C-tube (Miltenyi, Cat#130-093-237) with 3 mL cRPMI. The tubes were loaded onto the GentleMACS dissociator and run with the appointed program. The dissociated splenocytes were placed on top of 70 µM cell strainers (Corning, Cat#43,175) and 50 mL Falcon tube and filtered through. Cells were washed twice by resuspension in cRPMI and centrifugation at 1500 rpm for 5 min at 4 °C. The cells were finally dissolved in pure FCS and 10% DMSO (dimethyl sulfoxide, Sigma-Aldrich, Cat#C6164), and 1 mL was aliquoted into cryo tubes and cryopreserved at − 180 °C.

### Generation of pH-2 monomers

All peptides for pH-2 multimer libraries were purchased from Pepscan (Pepscan Presto BV, Lelystad, Netherlands) or TAG Copenhagen (Frederiksberg, Denmark) and dissolved to 10 mM in 100% DMSO. A full list of peptides used in the study can be found in Supplementary Table 2. Specific peptide binding to each H-2 molecule was in silico predicted via NetH2pan^22^, as described in the previous section on UV ligand design. The five different murine H-2 monomers and their corresponding peptides were diluted in Phosphate Buffered Saline (PBS). Monomers (50–100 µg/mL final concentration) were added to the corresponding peptide (100–200 µM final concentration) and placed under a 366 nm UV light for 1 h at room temperature, to replace the UV ligand with the peptide of interest. A corresponding UV ligand control for each H-2 allele was in parallel incubated at RT on the bench.

### Generation of PE-conjugated pMHC tetramers and detection of pH-2-specific T cells

Tetramers were used to screen for T cell recognition against the specific peptide or irrelevant peptides in immunized or naïve mice. PE-labeled streptavidin (phycoerythrin, Biolegend, Cat#405,203, 0.2 mg/mL/100 µl pMHC) was loaded with exchanged pH-2 (100 µg/mL monomer and 200 µM peptide) for 30 min on ice. 500 µM D-biotin (Avidity, Cat#BIO200) was added and incubated for 20 min on ice, and the final product was stabilized using a 10 × freezing media (PBS with 0.5% Bovine Serum Albumin (BSA) and 5% glycerol) and stored at − 20 °C.

Splenocytes from immunized and naïve mice were thawed and washed twice in cRPMI. 3–4 × 106 cells were plated into individual wells in a V-bottom 96 well plate and washed with FACS buffer (PBS and 2% FCS). Cells were treated with 0.5 µl Fc receptor block (Biolegend, Cat#101,301)for 15 min to block unspecific binding. Tetramers were centrifuged prior to use. 1 µl of each pH-2 tetramer specificity was added to the corresponding sample with 49 µl BV buffer (BD, Cat# 566,385) and 0.5 nM Dasatinib and incubated for 15 min at 37 °C. Cells were stained with an antibody mix (CD3-FITC: Biolegend Cat#100,306, CD8-BV480: BD Cat#566,096 and the dead cell marker LIVE/DEAD Fixable Near-IR: (Thermo Fischer, Cat#L10119) for 30 min at 4 °C. After two washes in FACS buffer, the cells were either filtered using blue cap FACS tubes (Falcon, Cat#352,235) and acquired directly on the flow cytometer or fixed using 1% filtered paraformaldehyde (Thermo Fischer, Cat# 11,400,580) for 1–24 h before acquisition.

### Generation of large p*H-2 multimer library and detection of pH-2-specific T cells

A p*H-2 library was used to screen for T cell recognition against the specific pH-2 or irrelevant pH-2 specificities in immunized or naïve mice. The technique is described in previous publications^4^. Briefly, different pH-2 complexes (50 µg/mL monomer and 100 µM peptide) were prepared (Supplementary Table 1) and coupled to PE-labeled dextran backbones loaded with a unique DNA barcode). Splenocytes from immunized and naïve mice were thawed and washed twice in cRPMI. 3–4 × 106 cells were plated into individual wells in a 96 well plate and washed with Barcode-Cytometry buffer (PBS with 0.5% BSA, 100 µg/mL herring DNA, 2 mM EDTA). A pool consisting of 1.5 µl of each pH-2 specificity was gathered and filtered through a 10 kDa cut-off membrane filterSartorius). Cells were stained with the pool of the pH-2-multimers and with an antibody mix (as described in the previous section) before FACS acquisition and sorting. pH-2 multimer-specific T cells were sorted based on single, live, CD3+ CD8+ PE + cells. The sorted cells labeled with DNA barcoded pH-2 multimer reagents were centrifuged and pellets stored at − 20 °C. The number of sorted cells can vary substantially depending on the sample and composition of such. It is recommended to sort as many cells as possible, minimally 20 pH-2 multimer-specific T cells. Most samples will result in the sorting of 100–20.000 cells. The DNA barcodes that were present in the samples at sorting were amplified along with triplicate full library baseline samples for comparison (aliquot of pH-2 multimer reagent pool). The amplified product was purified using a QIAquick PCR Purification kit (Qiagen, Cat#28,104), sequenced (IonTorrent, Primbio), and data were processed by the publically accessible software Barracoda, developed at DTU (http://www.cbs.dtu.dk/services/barracoda). Barracoda calculates the total reads and clonally-reduced reads for each DNA barcode (relating to its coupled pH-2 specificity). Log2 fold changes in read counts linked to a given sample, related to the mean read counts, are compared to the baseline samples and estimated with normalization factors determined by the trimmed mean of the M-values method. False-discovery rates (FDRs) were estimated using the Benjamini–Hochberg method. A p-value was calculated based on the Log2 fold change distribution, determining the strength of the signal compared to the input, and *p* < 0.001, corresponding to FDR < 0.1%, is established as the significance level determining a T cell response.

### Flow cytometry

All flow cytometry experiments were carried out on LSR-Fortessa and Melody instruments (BD Biosciences). Data were analyzed in FlowJo version 10.7.1 (TreeStar, Inc.).

### Statistical analysis

GraphPad Prism 7 for Windows was used for graphing, statistical analyses, and tools.

### Ethical approval

All experiments were conducted according to the research ethics guidelines of the Technical University of Denmark, all animal experiments were approved by the Danish National Animal Welfare Committee (dyreforsoegstilsynet, approval no. 2020-15-0201–00,748). The studies comply with the ARRIVE guidelines.

### Supplementary Information

Supplementary Information.

## Data Availability

The datasets generated and/or analyzed in this study are available from the corresponding author upon request.
