# Supplementary material for: Validation of novel conditional ligands and large-scale detection of antigen-specific T cells for H-2Dd and H-2Kd
Source: Sci Rep. 2024 May 29;14:12292. doi: 10.1038/s41598-024-62938-8 (PMC11136991; doi:10.1038/s41598-024-62938-8)
Supplement: Supplementary file 1 — Supplementary Information. [file 41598_2024_62938_MOESM1_ESM.pdf]

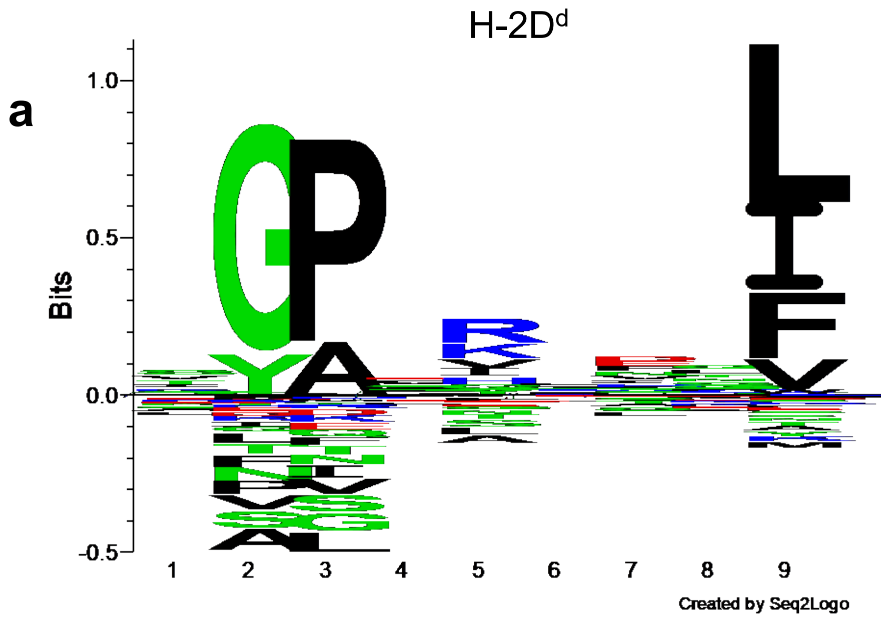

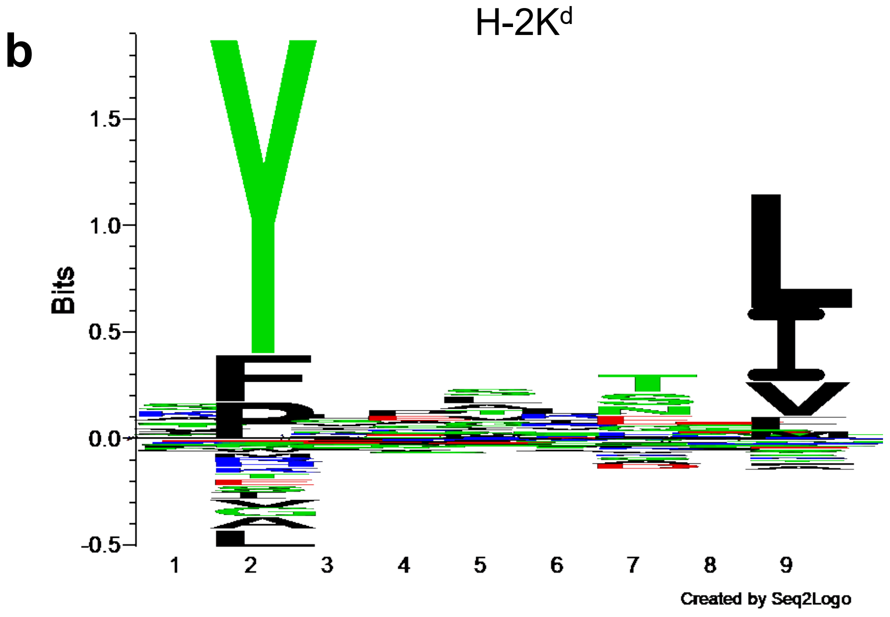

Supplementary Figure 1.
MHC allele motif view based on eluted naturally presented ligands to visualize positional amino acid binding preferences of murine MHC alleles H-2D^d (a) and H-2K^d (b). Logos created by Seq2Log (http://www.cbs.dtu.dk/services/NetMHCpan/logos_ps.php).

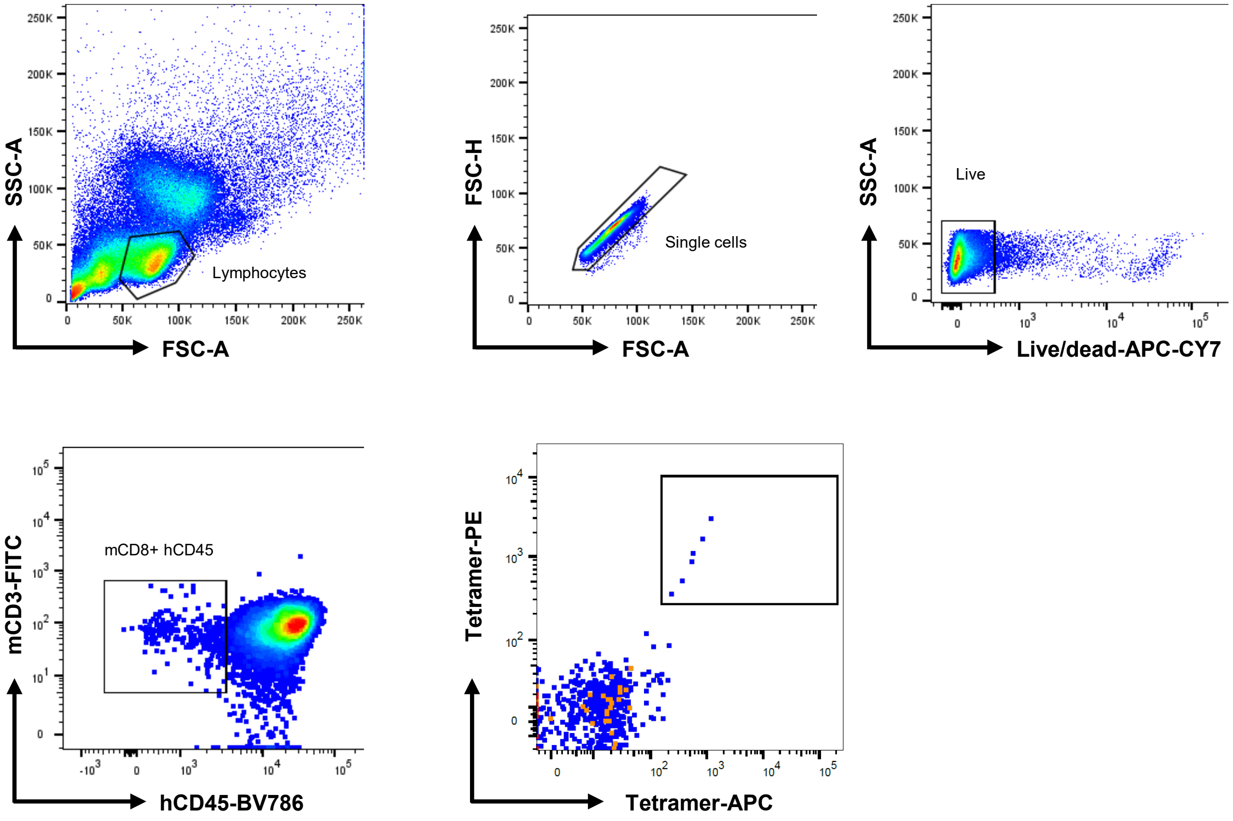

Supplementary Figure 2.

FACS gating strategy for PE-APC labeled pH-2 tetramer stainings of the H-2k^{b} antigen-specific sample. Samples were acquired on BD LSR Fortessa and analyzed by Flowjo 10.7.1.

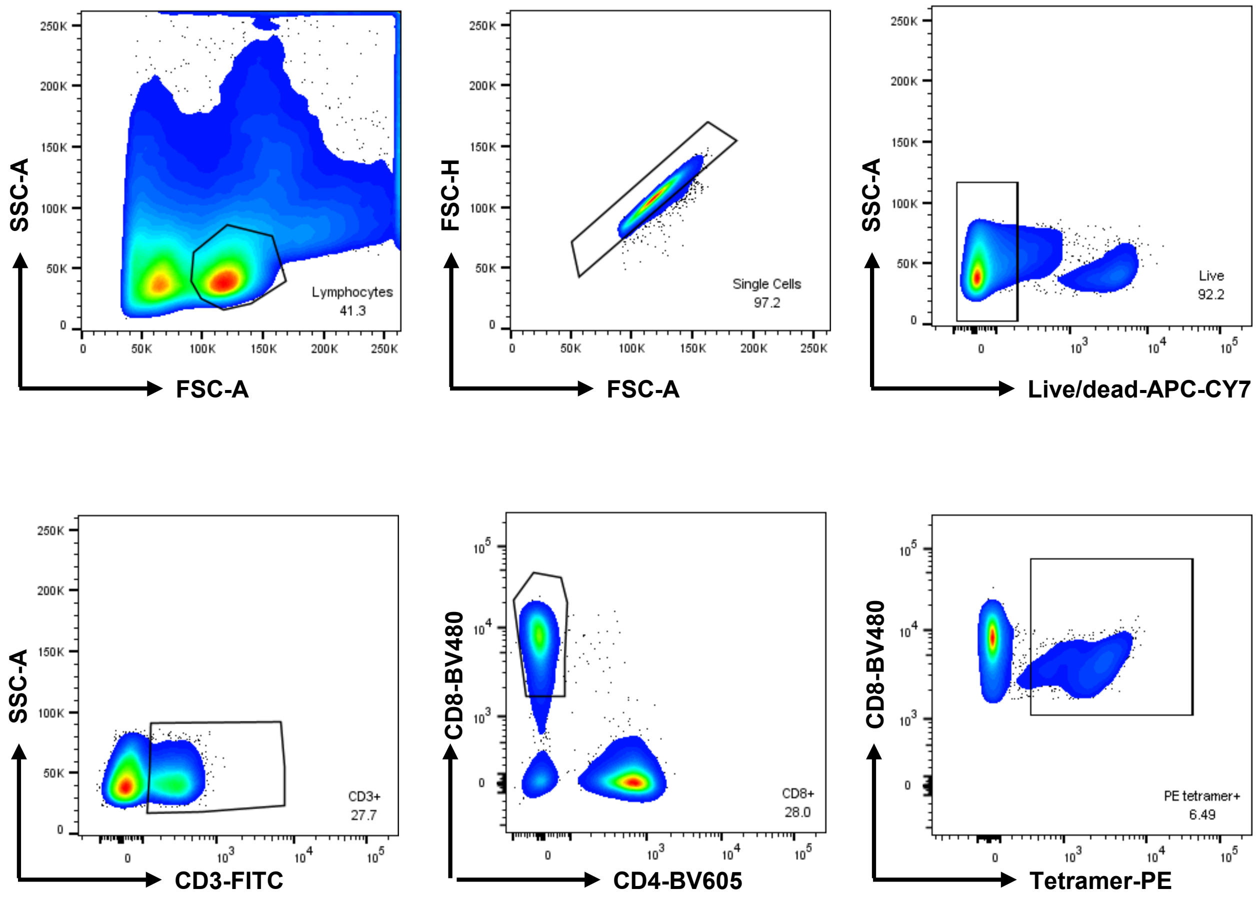

Supplementary Figure 3.
FACS gating strategy for PE-labeled pH-2 tetramer stainings of the H-2L^{d} antigen-specific sample. Samples were acquired on BD LSR Fortessa and analyzed by Flowjo 10.7.1.

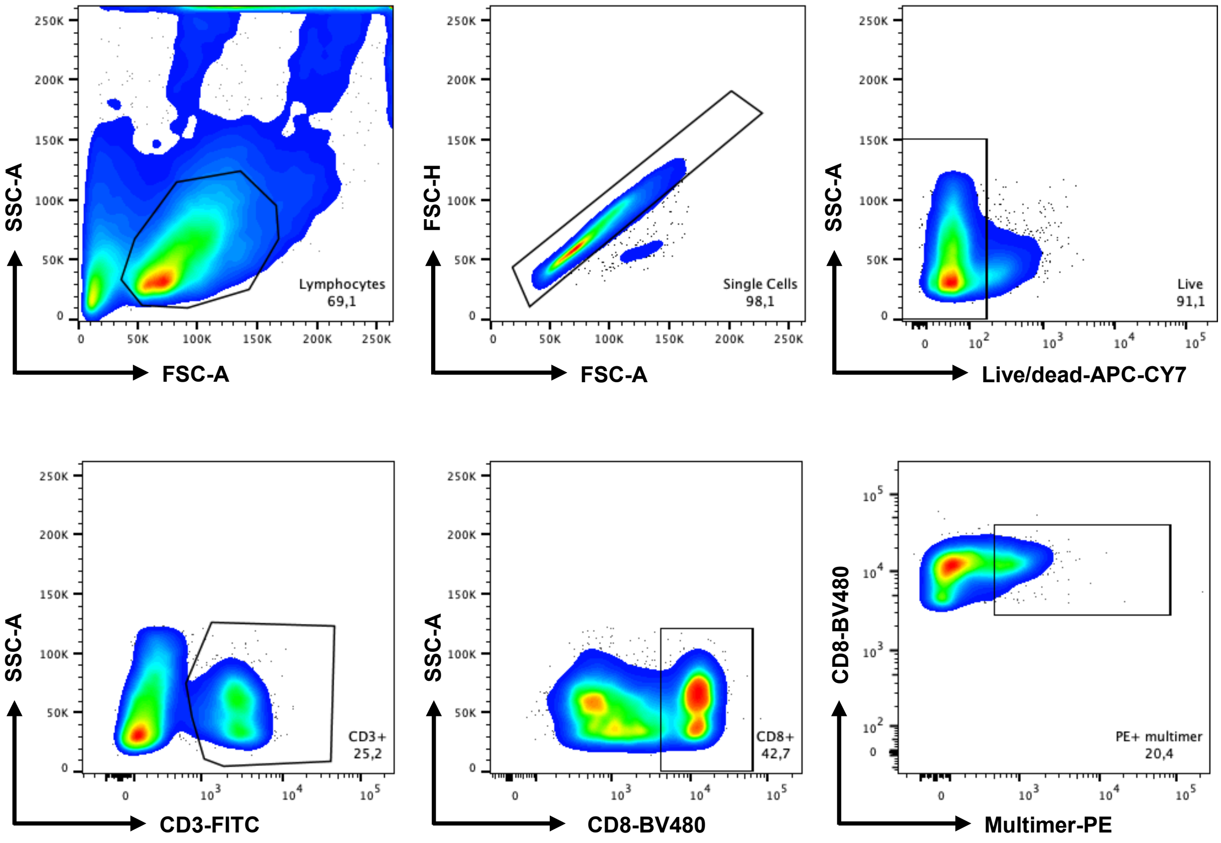

Supplementary Figure 4.
FACS gating strategy for PE-labeled DNA barcode pH-2 stainings of the H-2K^d antigen-specific sample. Samples acquired on BD Melody and analyzed by Flowjo 10.7.1.

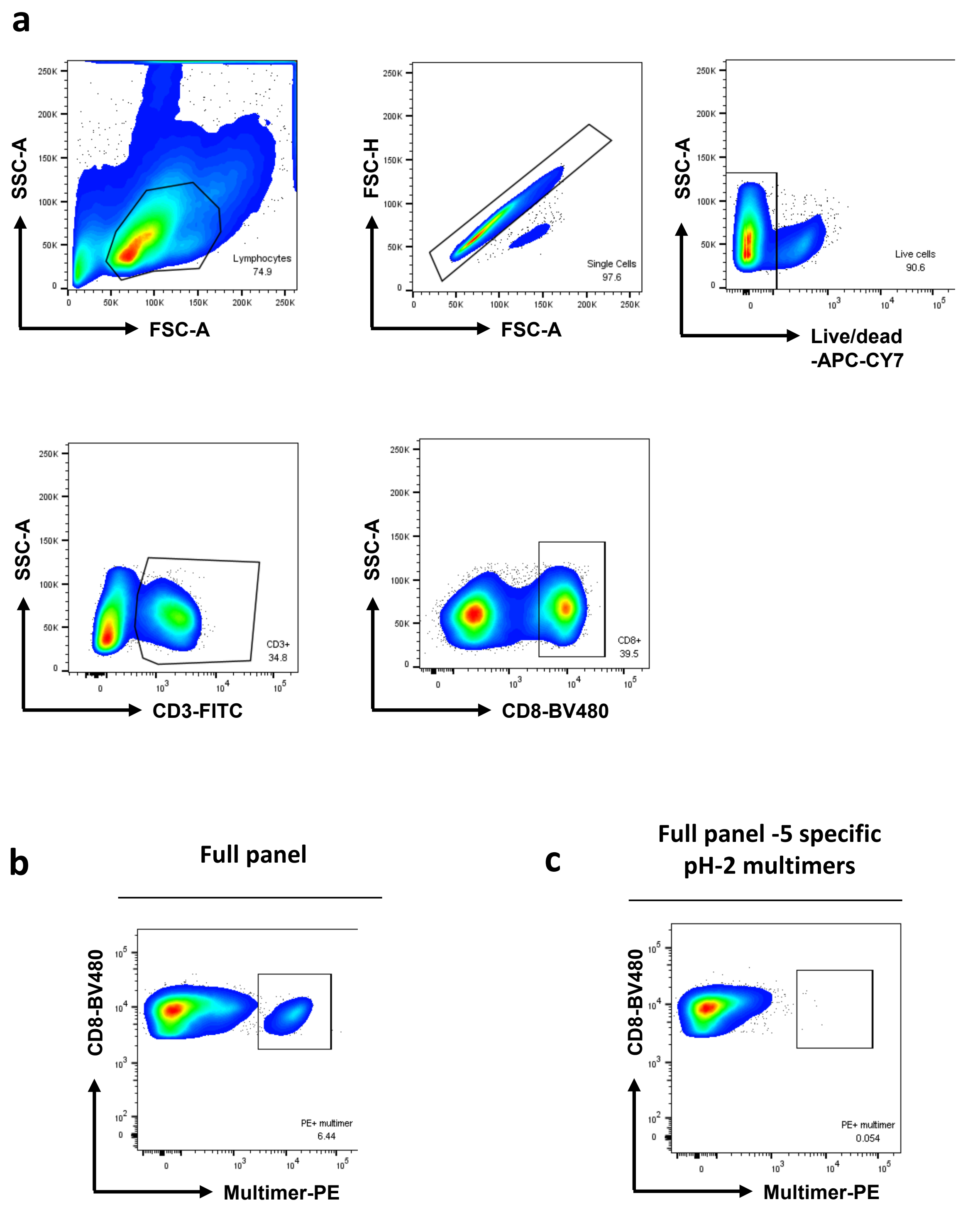

Supplementary Figure 5.
FACS gating strategy (a) for PE-labeled DNA barcode pH-2 stainings of a spike-in+ mix of antigen specific samples with full pH-2 panel (b) or spike-in- mix of antigen specific samples with full panel minus the five specific pH-2 multimers (c). Samples acquired on BD Melody and analyzed by Flowjo 10.7.1.

Add gatting strategy for figure 2 here: H-2Kb results

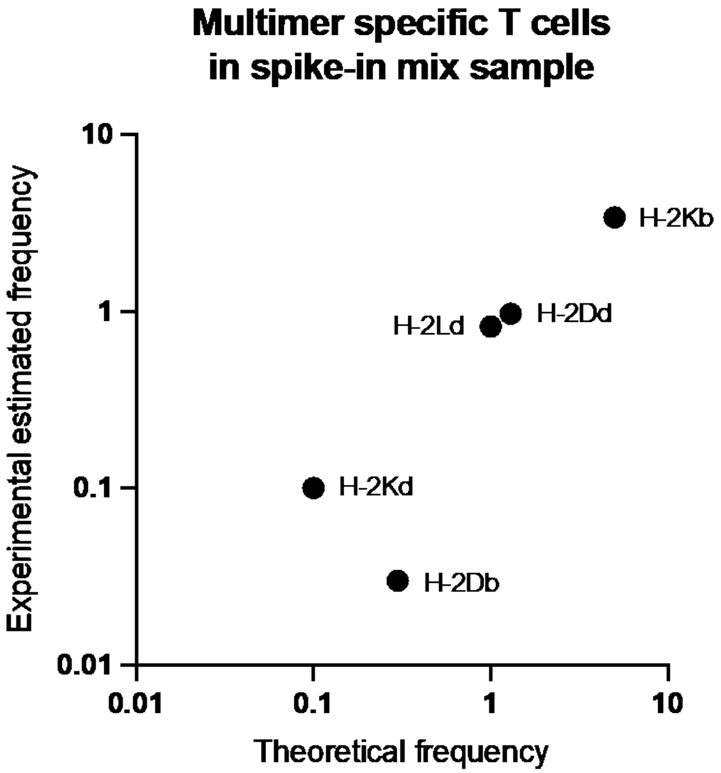

Supplementary Figure 6.
Comparison of 1) *Theoretical frequency* (x-axis): calculation after diluting individual pH-2 antigen specific samples into each other to create the spike-in mix, and 2) *Experimental estimated frequency* (y-axis): calculation: fraction of PE+ CD8+ T cells (%) multiplied by count fraction (%) of the specific DNA barcode out of the entire library of DNA barcodes after sequencing of the sorted cells.

Supplementary table 1.
Protein Yield of murine MHCI after *in-vitro folding*, biotinylation, and purification

	refolding volume in ml	Final yield in mg
**H-2Db**	200	4.2
	500	12.5
**H-2Dd**	400	1.2
	200	1.0
**H-2Kb**	400	5.1
	100	2.1
**H-2Kd**	200	1.0
	500	3.4
**H-2Ld**	400	2.9
	200	1.3

Supplementary table 2.
All peptides used to generate p*H-2 multimers during this study.

Peptide	Sequence	H-2 allele	%rank score	P*H-2 tetramers	DNA barcode-labeled p*H-2 multimers
**HPV16 $E7_{49-57}$**	**RAHYNIVTF**	**H-2Db**	**0.0592**	**X**	**X**
**MC38_1**	AKKTNAGVL	H-2Db	2.2319	X	X
**MC38_2**	ASKSLPATYI	H-2Db	5.3522		X
**MC38_3**	ASTTSNSASTF	H-2Db	7.0568		X
**MC38_4**	ATCTPREPV	H-2Db	6.3137		X
**MC38_5**	EELNNEDYY	H-2Db	5.3715		X
**MC38_6**	EELNNEDYYSL	H-2Db	5.5626		X
**MC38_7**	FASMHRHGV	H-2Db	4.5225		X
**MC38_8**	FQTLQAKFL	H-2Db	4.8082		X
**MC38_9**	FSNQLEGKWAL	H-2Db	7.8436		X
**MC38_10**	HGVVNQLGG	H-2Db	8.3393		X
**MC38_11**	HGVVNQLGGV	H-2Db	3.2544		X
**MC38_12**	HGVVNQLGGVF	H-2Db	2.7805		X
**MC38_13**	HMMKNQVMR	H-2Db	7.9355		X
**MC38_14**	HNPSANSIIV	H-2Db	3.3518		X
**MC38_15**	KAKKTNAGVL	H-2Db	2.3657		X
**MC38_16**	MALFSDVLL	H-2Db	1.5439		X
**MC38_17**	NQLEGKWALL	H-2Db	7.6336		X
**MC38_18**	QMSNPGLF	H-2Db	7.872		X
**MC38_19**	QQQAHRLTTL	H-2Db	7.6088		X
**MC38_20**	RTIRTGHGVV	H-2Db	7.7953		X
**MC38_21**	SRALEDRNRV	H-2Db	1.2572		X
**MC38_22**	STTSNSAST	H-2Db	2.3645		X
**H-2 Db UV-control**	ASNEN-**J**-ETM #	H-2Db	0.0026		X
**HIV $Env_{311-320}$**	**RGPGRAFVTI**	**H-2Dd**	**0.0081**	**X**	**X**
**CT26_1**	KADCLFTHM	H-2Dd	0.3119	X	X
**CT26_2**	VSPKDIQLTI	H-2Dd	0.3822		X
**CT26_3**	NNPSFPTGKM	H-2Dd	0.4959		X
**CT26_4**	VNPAVKIVFL	H-2Dd	0.4907		X
**CT26_5**	GGFQEFNFI	H-2Dd	0.1152		X
**CT26_6**	MGPPGGFQEF	H-2Dd	0.4322		X
**CT26_7**	SGPSYATY	H-2Dd	0.0733		X

**CT26_8**	PSGPSYATYL	H-2Dd	0.1128		X
**CT26_9**	GPSYATYL	H-2Dd	0.1957		X
**CT26_10**	SGPSYATYLQ	H-2Dd	0.2034		X
**CT26_11**	APSGPSYATYL	H-2Dd	0.273		X
**CT26_12**	YGFKEETI	H-2Dd	0.391		X
**CT26_13**	ENPETSVSM	H-2Dd	0.3165		X
**CT26_14**	VNDELWATI	H-2Dd	0.2688		X
**CT26_15**	TGPYVMMI	H-2Dd	0.3828		X
**CT26_16**	TSPPMPIL	H-2Dd	0.1355		X
**CT26_17**	DAGPTQFTTPL	H-2Dd	0.3012		X
**CT26_18**	TCPYCFQLL	H-2Dd	0.1767		X
**CT26_19**	NIVQCFIAL	H-2Dd	0.4446		X
**CT26_20**	TMPTFPHLV	H-2Dd	0.2915		X
**CT26_21**	IMPPVGTDL	H-2Dd	0.3747		X
**CT26_22**	STGKLLVAL	H-2Dd	0.1753		X
**H-2Dd UV-control**	RGPGRA-**J**-VTI#	H-2Dd	0.0347		X
**Influenza $HA_{518\text{-}526}$**	**IYSTVASSL**	**H-2Kd**	**0.0077**	**X**	**X**
**CT26_23**	IYQRKSDGI	H-2Kd	0.1259	X	X
**CT26_24**	IYSRTTDVL	H-2Kd	0.077		X
**CT26_25**	EYPSPSPL	H-2Kd	0.4866		X
**CT26_26**	KYLSVQSQL	H-2Kd	0.0029		X
**CT26_27**	WKYLSVQSQL	H-2Kd	0.1569		X
**CT26_28**	KYLSVQSQLF	H-2Kd	0.2979		X
**CT26_29**	YLSVQSQL	H-2Kd	0.3121		X
**CT26_30**	IYVALLRVM	H-2Kd	0.0464		X
**CT26_31**	AYVNAIEKI	H-2Kd	0.0074		X
**CT26_32**	IYLESVAIM	H-2Kd	0.0411		X
**CT26_33**	SIYLESVAIM	H-2Kd	0.2374		X
**CT26_34**	SYIETLPKAI	H-2Kd	0.0451		X
**CT26_35**	SSYIETLPKAI	H-2Kd	0.2745		X
**CT26_36**	SYIETLPKAIK	H-2Kd	0.4146		X
**CT26_37**	QFENLAQQL	H-2Kd	0.3605		X
**CT26_38**	GFVVGTMTL	H-2Kd	0.2631		X
**CT26_39**	SWDTSKKNL	H-2Kd	0.3546		X
**CT26_40**	NYVFKAAML	H-2Kd	0.3888		X
**CT26_41**	KYNDTPQSL	H-2Kd	0.0041		X
**CT26_42**	EKYNDTPQSL	H-2Kd	0.2982		X
**CT26_43**	KYNDTPQSLR	H-2Kd	0.4523		X
**CT26_44**	QEKYNDTPQSL	H-2Kd	0.4625		X
**H-2Kd UV-control**	IYSTV-**J**-SSL#	H-2Kd	0.0091		X

**LCMV NP$_{118-126}$**	**RPQASGVYM**	**H-2Ld**	**0.0586**	**X**	**X**
**CT26_45**	IPRDLFEGEL	H-2Ld	0.2826	X	X
**CT26_46**	SPNTSFASDGF	H-2Ld	0.4647		X
**CT26_47**	IGQMLQTHF	H-2Ld	0.009		X
**CT26_48**	SPKDIQLTI	H-2Ld	0.3472		X
**CT26_49**	IDPLALMQAI	H-2Ld	0.2198		X
**CT26_50**	DPLALMQAI	H-2Ld	0.4767		X
**CT26_51**	LEHLNIVTF	H-2Ld	0.4745		X
**CT26_52**	VPDGGAEHI	H-2Ld	0.2945		X
**CT26_53**	FPYANVAFPHL	H-2Ld	0.1685		X
**CT26_54**	FPYANVAF	H-2Ld	0.241		X
**CT26_55**	LPNILTKL	H-2Ld	0.274		X
**CT26_56**	SPYVYEIYMTF	H-2Ld	0.0125		X
**CT26_57**	SPKYTLRSHF	H-2Ld	0.0092		X
**CT26_58**	WSPKYTLRSHF	H-2Ld	0.1308		X
**CT26_59**	SPKYTLRSHFD	H-2Ld	0.2234		X
**CT26_60**	KEFPLFLLF	H-2Ld	0.3881		X
**CT26_61**	FPLFLLFL	H-2Ld	0.4722		X
**CT26_62**	IPILEMQF	H-2Ld	0.1054		X
**CT26_63**	MPEVIPILEM	H-2Ld	0.2826		X
**CT26_64**	LPVKDELLCQL	H-2Ld	0.4274		X
**CT26_65**	QPMAVSRFF	H-2Ld	0.1133		X
**CT26_66**	QPTSPPMPI	H-2Ld	0.0991		X
**H-2Ld UV-control**	YPNVNIH-J-F#	H-2Ld	0.01		X
**Ovalbumin$_{257-264}$**	***SIINFEKL***	***H-2Kb***	**0.0027**	***X***	***X***
**MC38_37**	ASMHRHGVV	H-2Kb	5.5919	X	X
**4T1_74**	MIIAYAYRGNL	H-2Kb	0.917		X
**4T1_75**	KVLHQNRL	H-2Kb	0.9447		X
**4T1_76**	KIRPFLKAHL	H-2Kb	1.0062		X
**4T1_77**	TVFNSRFL	H-2Kb	1.092		X
**4T1_78**	RFFYMPRF	H-2Kb	1.314		X
**4T1_86**	CLLAHALNL	H-2Kb	1.7428		X
**4T1_87**	KTLTLEAI	H-2Kb	1.7522		X
**4T1_88**	STWKRAVM	H-2Kb	1.7522		X
**4T1_89**	AGFSSFQKLR	H-2Kb	1.8001		X
**4T1_90**	KTWPQVAPM	H-2Kb	1.8543		X
**4T1_229**	SPPRFFYM	H-2Kb	0.4917		X
**4T1_231**	TGIINHPLL	H-2Kb	0.3699		X
**4T1_233**	HMFLFGRL	H-2Kb	0.0203		X

**4T1_236**	IYHYVLNSM	H-2Kb	1.4979		X
**4T1_238**	SLITYIGL	H-2Kb	0.0436		X
**4T1_241**	RSGQSYELL	H-2Kb	1.6977		X
**4T1_244**	TGPPASRL	H-2Kb	0.5221		X
**4T1_249**	NAVMHTTSI	H-2Kb	1.6097		X
**4T1_251**	KVVTLHTKL	H-2Kb	0.1133		X
**4T1_252**	SGQSYELL	H-2Kb	0.5583		X
**4T1_258**	ISPTIIDL	H-2Kb	0.7001		X
**4T1_289**	RSPLFFIKC	H-2Kb	0.6648		X
**H2-Kb UV-control**	FAPGNY-**J**-AL#	H-2Kb	0.3095		X

"- J-"was replaced with "X" while writing as an input for rank calculation in NetH2pan
